# University of Maryland, Dental Department

**Published:** 1884-06

**Authors:** 


### University of Maryland, Dental Department.

The second annual commencement of this department, in connection with the seventy-seventh of the University, was held March 14, 1884, at the Academy of Music, Baltimore. After the reading of the mandamus the names of the graduates were announced by Prof. F. J. S. Gorgas, Dean of the Dental Department.

The "Degree of Doctor of Dental Surgery" was conferred upon the following gentlemen by Hon. S. Teackle Wallis, L.L.D., Provost of the University:

| NAME. | STATE. |
|---|---|
| Theodore W. Albright | New York. |
| James B. Bigham | S. Carolina. |
| Chas. B. Blubaugh, M.D. | W. Virginia. |
| Wilbur C. Bressler | Pennsylva. |
| Henry H. Boswell | Maryland. |
| John H. Brown | Ohio. |
| George Buttler, Jr. | New Jersey. |
| John M. Comegys | Tennessee. |
| George V. Copp | Virginia. |
| Almond J. Cutting | Massach'ts. |
| Isaac H. Davis | Maryland. |
| Watson E. Dorchester | New York. |
| Richard D. Evans | S. Wales, G. B. |
| Claus Henry Filter | Germany. |
| Nathan E. Foote | New York. |
| Frank C. Gallup | Connecticut. |
| James Edwin Harris | Maryland. |
| S. Dwight Hodge | Vermont. |
| R. Dallas Kibler | Virginia. |
| W. S. Killingsworth | South Carolina. |
| Chas. J. Ladson | Dist. Columbia. |
| Clarence E. Lemley | Virginia. |

| NAME. | STATE. | NAME. | STATE. |
|---|---|---|---|
| Wm. Edward Lewis | Florida. | A. LaF'y'te Stratford | North Carolina. |
| Job B. Mallott | Pennsylvania. | Reading B. Swindell | North Carolina. |
| Charles H. McDowell | North Carolina. | John E. Taggart | Vermont. |
| Semoney J. Minghini | West Virginia. | Robert R. Vaughan | Missouri. |
| S. Latimer Phillips | Virginia. | R. van der Hoppe | Austria. |
| George E. Purnell | Maryland. | Joseph T. Wayman | Virginia. |
| Nelson T. Shields | Texas. | Elmer J. Wisherd | Maryland. |

The valedictory address was delivered by Hon. J. Randolph Tucker, member of Congress from Virginia.

The number of matriculates for the session 1883–84 was eighty-six, all of whom were dental students.

---

*To the Faculties of the Dental Colleges of the United States:*

At an informal conference of the Deans of the Pennsylvania College of Dental Surgery, the Philadelphia Dental College, the Dental Department of the University of Pennsylvania, and the Baltimore College of Dental Surgery, it was decided to invite a meeting of the faculties (or their representatives) of all the dental colleges in the United States at the Sturtevant House, New York City, on Monday, August 4, 1884, for the purpose of adopting a uniform standard of graduation, etc.

(*Signed.*) R. B. WINDER,
JAMES TRUMAN,
J. E. GARRETSON,
C. N. PEIRCE.

The above notice and invitation has just come to hand. It is a movement in the right direction, and it is to be hoped that all of the dental colleges in the country will be represented, and come prepared to take some definite action for the promotion of dental education in our country. By establishing uniformity of requirements for entrance and for graduation a great point would be gained. In these respects there should be no competition. It is desired that the utmost harmony should prevail, and it is to be hoped that all colleges will co-operate.

